# Defining medical liability when artificial intelligence is applied on diagnostic algorithms: a systematic review

**DOI:** 10.3389/fmed.2023.1305756

**Published:** 2023-11-27

**Authors:** Clara Cestonaro, Arianna Delicati, Beatrice Marcante, Luciana Caenazzo, Pamela Tozzo

**Affiliations:** Legal Medicine Unit, Department of Cardiac, Thoracic, Vascular Sciences and Public Health, University of Padua, Padua, Italy

**Keywords:** artificial intelligence, diagnostic algorithm, medical liability, regulation, systematic review

## Abstract

Artificial intelligence (AI) in medicine is an increasingly studied and widespread phenomenon, applied in multiple clinical settings. Alongside its many potential advantages, such as easing clinicians’ workload and improving diagnostic accuracy, the use of AI raises ethical and legal concerns, to which there is still no unanimous response. A systematic literature review on medical professional liability related to the use of AI-based diagnostic algorithms was conducted using the public electronic database PubMed selecting studies published from 2020 to 2023. The systematic review was performed according to 2020 PRISMA guidelines. The literature review highlights how the issue of liability in case of AI-related error and patient’s damage has received growing attention in recent years. The application of AI and diagnostic algorithm moreover raises questions about the risks of using unrepresentative populations during the development and about the completeness of information given to the patient. Concerns about the impact on the fiduciary relationship between physician and patient and on the subject of empathy have also been raised. The use of AI in medical field and the application of diagnostic algorithms introduced a revolution in the doctor–patient relationship resulting in multiple possible medico-legal consequences. The regulatory framework on medical liability when AI is applied is therefore inadequate and requires urgent intervention, as there is no single and specific regulation governing the liability of various parties involved in the AI supply chain, nor on end-users. Greater attention should be paid to inherent risk in AI and the consequent need for regulations regarding product safety as well as the maintenance of minimum safety standards through appropriate updates.

## 1 Introduction

The application of artificial intelligence (AI) in medical field represents an emerging reality and the use of machinery that assist the professional operators both in the executive and in the diagnostic phases is increasingly frequent in hospitals (1). AI systems are currently destined to intervene on the treatment process both in the diagnostic and therapeutic phase, allowing the healthcare professional to reach, on the one hand, more certain and precise diagnoses, and on the other, surgical therapies, more effective and less invasive (2). The usefulness of AI has been described in radiology, because of its potential to recognize complex patterns and to provide quantitative assessments, and in oncology, as improving the prediction of outcomes, and nowadays its application outspreads to a large part of clinical settings (3, 4). Beside clinical advantages, the use of AI through risk stratification models can be useful to optimize resource plan and allocation (5). In the context of COVID-19 pandemic, opportunities to the AI research community emerged, as evidenced by the speed of production and dissemination of scientific works on this issue (6). Currently, the advantages of AI systems in the patient’s healthcare are widely affirmed, whereas the legal implications are still discussed (7). Since operators are not allowed to understand and verify the logical processes which drive the machine to the results, human–machine interaction in actions and omissions, especially when autonomous choices are taken by AI systems, poses relevant issues of liability in the event of damage to third parties. New technological approaches introduce new realities that might be unlikely to fit within the solid edges of the current law.

In this context, a comprehensive trace of present views about critical issues and in particular about liability related to the application of AI algorithms is essential in order to consider possible solutions.

## 2 Methods

In May 2023, one of the authors (CC) performed a review of the literature using the public electronic database PubMed. To ensure that no studies were missed, initially no temporal limits were set. The following search phrase was used: “medical liability diagnostic algorithm.” The research returned 96 articles. The same author repeated the review in June 2023, using the same search phrase, and found 97 articles.

Inclusion criteria: articles published between 2020 and 2023, dealing with critical issues, in particular liability, related to the use of AI and AI algorithms in medicine were included.

Exclusion criteria: articles not written in English were excluded.

The study selection was performed basing on titles and on abstracts. Overall, 29 articles were sought for retrieval. A full-text reading of retrieved articles was performed. After excluding articles whose full texts did not fulfill the abovementioned criteria, 23 articles were finally included in the review.

**FIGURE 1:**
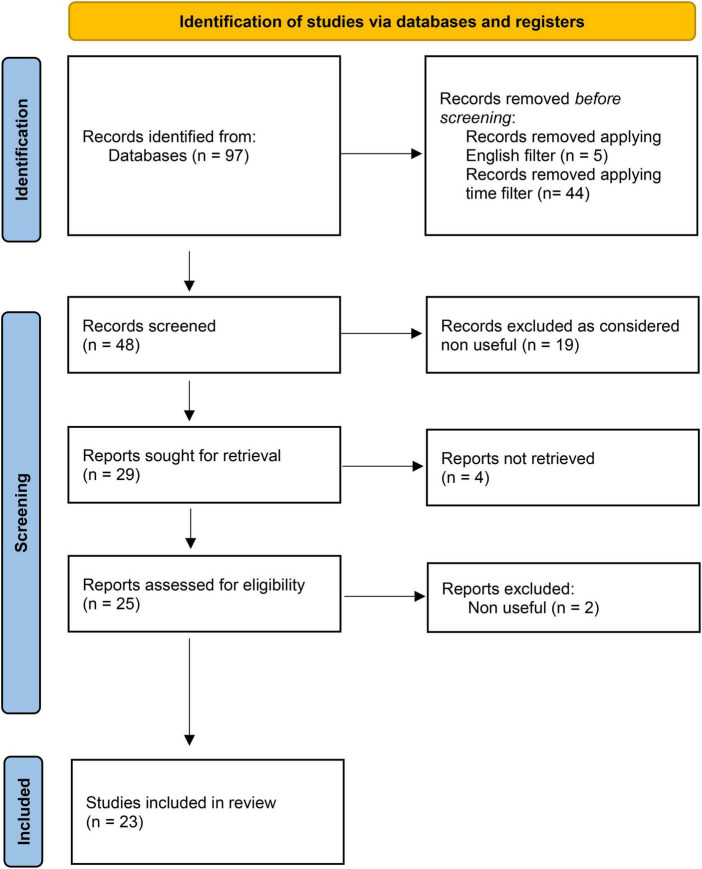
PRISMA flow-chart adapted from Page et al. (45).

The number of articles included and excluded was registered in a PRISMA flow chart (Figure 1).

**FIGURE 2:**
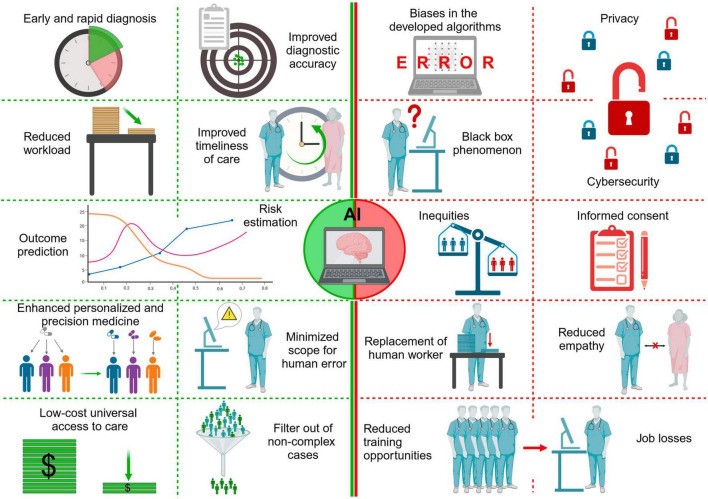
Graphical representation of the pros and cons of using artificial intelligence in diagnostic pathways (created with BioRender.com).

**FIGURE 3:**
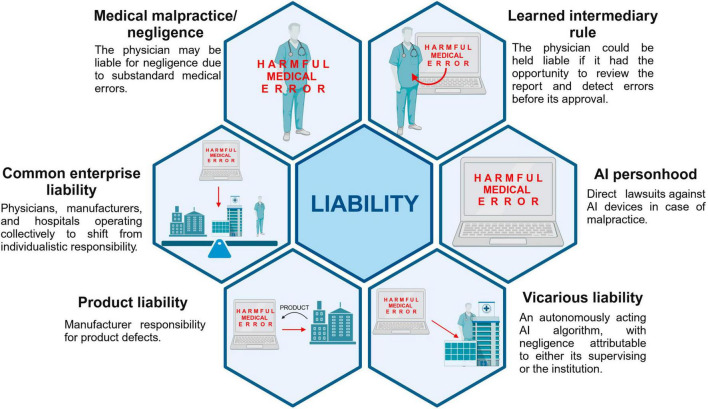
Possible approaches to liability issue (created with BioRender.com).

Data concerning advantages of AI, critical issues and liability issue were collected and summarized in Table 1 and Figures 2, 3.

**TABLE 1:** Summary of results concerning advantages of AI, critical issues, and liability issue.

References	Advantages of AI	Critical issues	Liability
Bhardwaj (1)	Enhance diagnostic modalities. Improve therapeutic interventions. Increase workflows. More accurate prediction. Genome interpretation. Improve triage.	Shift away from traditional in-person interaction. Added workload during the initial phase. Increase false-positive and -negative alerts. Paucity of cost-benefit data. Privacy.	Detrimental outcomes should be apportioned between developer, interpreter, healthcare providing, and patient.
Chung et al. (8)	More accurate interpretation. Screening tool. Improve quality of care. Early diagnosis.	Blindly trust of unexperienced clinicians. Systemic bias. Exacerbation of disparities. Cybersecurity. Privacy. Standardization. Interpretation and incorporation of results in clinical practice.	Manufacturing companies should clearly state indications and possible adverse effects in using an AI algorithm.
Emiroglu et al. (9)	Rapid diagnosis. More detailed evaluation. Examination of a larger number of patients.	Replacement of individual’s job.	Concerns about medical errors and allocation of responsibility.
Mezrich (10)	Handle increased quantities of patient imaging. Increase accuracy, fastness. Role of second set of eyes.	Replacement of radiologists. Physician–patient relationship. Standard of care. Extra work for physicians.	Types of tort law that may be implied are medical malpractice, vicarious liability, products liability (with learned intermediary exception). Legal handling will be based on the degree of autonomy of AI software. AI personhood as solution. Possible approach similar to National Vaccine Compensation Program.
Mezrich (11)	Greater volume of imaging. Role of second set of eyes. Increase diagnostic accuracy and prevent negative outcomes.	Replace physicians. Additional work. Privacy.	Potential causes of action that may be implied include medical malpractice, vicarious liability, and products liability (with learned intermediary exception). Negligence could reflect a failure of programming, supervision, actions of physician or of the algorithm. AI personhood as solution. Possible approach similar to National Vaccine Compensation Program.
Vedantham et al. (12)	Reduce workload. Improve workflow. Improve timeliness of care. Accurate risk estimation.	Unreliability in case of out of distribution data.	AI preselection influence on radiologist’s ability to search: perceptual error of AI.
Harvey and Gowda (13)	Improve physician performance and minimize the scope for human error.	Amplification of racial and demographic disparities. Confounding variables. Privacy.	Radiologists, their employer in healthcare system and developers are subject to various legal theories of liability: radiologists will face medical professional liability under standard negligence principles, healthcare systems may encounter vicarious liability, AI developers may face product liability or negligence framework or strict liability.
Harvey and Gowda (14)	Aid in diagnosis.	Black box. Informed consent.	Absence of clinical AI related case law. If it can be demonstrated that the physician could have identified the finding missed by the AI, causation can be proved. If AI algorithms will be integrated into radiology standard of care, deviations from AI readout may indeed prompt liability. Healthcare systems could face vicarious liability. Nature of AI-developer’s liability is unclear.
Sung and Poon (15)	More accurate assessment and information. Personalized treatments. Improve treatment efficacy. Assist surgeons. Replace time-consuming and repetitive tasks. Less variations. Absence of fatigue. Assist interpretation. Predict clinical outcome.		A significant determinant is the degree of control. If the clinician or the AI developer is capable to prevent the accident, there is liability. When decisions are made by self-learned algorithms, without need of clinician approval, liability will be at least partially of the developer. Events caused by defects will likely be developers’ responsibility in product liability claim. Main relevant aspects are circumstances of accident (control and information) and conditions of AI device.
Pecqueux et al. (16)	Improve diagnostic accuracy. Reduce time spent on monotonous tasks. More precise and minimally invasive surgical techniques.	Applicability to controversial issues. Low ability to empathize and take into account patients’ emotions.	Cooperative effort of surgeons and assistive AI systems as a solution.
López et al. (17)	Reduce workload of health workers. Help to close gaps in healthcare and improve public health surveillance. Improve patient care, diagnosis, treatment, and public health efficiency.	Contextual bias. Inequality. Exacerbate disparities. Lack of understanding of AI by different stakeholders. Accountability in black box models. Privacy.	Unexplainably of black box algorithms and lack of clear policy and legal frameworks in low-middle income countries make liability a challenge.
Bazoukis et al. (18)	Support less-trained physicians. Filter out non-complex cases. Facilitation of screening and evaluation in areas with restricted access to medical expertise. Early disease detection. Increase diagnostic accuracy. Identification of new patterns. Personalized approach.	Exacerbation of inequities in health outcomes. Cybersecurity.	AI developers should report and investigate potential adverse events/system failure. Physicians have responsibility to use an algorithm as labeled.
Musacchio et al. (19)	Identification of risk factors. Identifying variables. Risk stratification. Personalized medicine. Reduce time spent collecting data. Reduce costs. Improve sensitivity and specificity of disease detection and diagnosis. Reduce human error in decision-making. Increase efficiency, reproducibility, and coverage of screening programs. Reduce obstacle to accessing and improving results. Early diagnosis and treatment. Better allocation of resources.	Heterogeneity of unstructured data. Quality and correct use of data. Privacy. Reduce doctors’ skills and job replacement. Difficulty in knowing and interpreting new analysis models.	
Channa et al. (20)	Earlier identification of at-risk patients. Earlier diagnosis. Improve patient care.	Privacy. Racial biases.	Primary care physicians using AI, not having specialized knowledge, should not be liable for incorrect result. AI creator or AI selling company should assume medical liability.
Abràmoff et al. (21)	Improve access to care. Increase accuracy. Lower costs.	Inappropriate biases. Non-appropriate use of patients’ data. Job losses. Transparency.	Creators of autonomous AI should assume liability for harms when the device is used properly and on-label and obtain medical malpractice insurance. Responsibility for proper use and maintenance of device remains with the providers. In case of assistive AI, the physician remains fully liable.
Pai and Pai (22)	Better diagnostic performance. Low-cost universal access to care.	Underperformance when there is no precedence. Holistic approach with human touch. Black box. Privacy. Biases. Job losses.	
Filipow et al. (23)	Prediction of clinical outcomes. Enrich personalized medicine.	Increase or create of health disparities among marginalized groups (inconsistencies in access to healthcare or under representation of some groups). Validation and replicability.	In the absence of clearer regulations about liability, the potential for implementation of decision-making tools is yet to be fully realized because of the high risk of erroneous prediction.
Nitiéma (24)	Enhance personalized medicine. Improve accuracy of diagnostic procedures. Promotion of precision medicine.	Replacement of human workers. Involvement of private companies. Reduced quality of medical care. Reduce importance of human touch and empathy. Biases in the developed algorithms. Ambiguity about role and tasks. Reduced equity and access to care.	Difficulties in apportioning liability (black box algorithms). Algorithms’ autonomy makes lawsuit against manufacturer challenging. Learned intermediary doctrine prevents patients from directly suing manufacturers. AI personhood as possible solution. AI user could purchase a liability insurance.
Neri et al. (25)	Impact on workflows, improving and automating acquisition protocols, appropriateness, structured reporting, and ability to interpret big data.	Black box. Automation bias. Lack of motivation to pursue career as radiologist. Lack of radiologist specialists. Reduce training opportunities. Transparency toward patients. Information.	Using AI, the radiologist is responsible for the diagnosis. The solution could be the creation of an ethical AI, subject to constant action control and to a vicarious liability, for which the producers must guarantee the users.
Chan (26)	Improve diagnosis and prognosis.	Black box. Exploitation of unknown and unreliable confounding variable. Generalizability. Difficult evaluation of product quality. Autonomy and informed consent.	Physicians, manufacturers, and hospitals should be considered a common enterprise for the purpose of liability, and they should be held jointly and strictly liable for harms caused by clinical AI systems.
Lang et al. (27)	Augment and extend the effectiveness of cardiologist in cardiovascular imaging.	Opacity in AI decision-making. Black box. Bias or non-generalizable patterns enshrined in machine learning. Intelligibility.	Question about the standard of care to which clinicians that use unexplainable models are subject. Question about causation (difficult to demonstrate that an alleged injury was caused by the breach of duty of care). Unexplainable AI likely to generate challenges in product liability. The failure to use or the reliance on AI might be considered a fault when best medical practice would suggest a different approach.
Jobson et al. (28)	Quick and accessible opinion.	Black box. Uncritical deference to AI results in both diagnosis and treatment. Information. Rate of false negatives/false positives. Privacy. Variable performance.	Answer to the liability issue depends on whether the software is used directly by patients or by clinicians to aid decision-making. Software developers and suppliers likely are to owe a duty of care to patients.
Patcas et al. (29)	Optimize processes and enrich care. Facilitate predictive and preventive medicine. Faster and more reliable healthcare. Improve personalized medicine. Reduce costs and increase accessibility.	Biases. Black box. Discrepancies with individual’s perception. Informed consent. Cybersecurity. Privacy. Intellectual proprietary right.	Question about the presence, in case of treatment based on AI-software’s incorrect prediction, of medical malpractice or product liability.

## 3 Results

### 3.1 Article type

Most of included articles consist in perspectives articles and reviews, whose construction in most of cases do not meet the criteria of systematic review (*n* = 1 systematic review, *n* = 1 scoping review). *N* = 2 surveys are included in the study.

### 3.2 Advantages of AI in medicine

Improvements in diagnostic accuracy and in personalized medicine represent frequently reported advantages of AI use in medicine. With regard to accuracy, in cardiovascular field, Chung et al. (8) report that “the use of AI-ECG algorithms for rhythm identification and ECG interpretation can be more accurate in interpretation” than current ECG software, and also affirm that AI-based ECG analysis has been proposed as an accurate screening tool in valvular disease field. In breast disease and cancer care area, the use of AI is thought to lead to rapid diagnosis and more detailed evaluation (9). It is important to note, with regard to computer-aided detection (CAD) software that, according to Mezrich (10, 11), these can increase radiologists’ accuracy and fastness, but they act as a “second set of eyes.” Consistently, Vedantham et al. (12) affirm that it is more likely that AI will be used as a second reader, and Harvey and Gowda (13) reports the greater effectiveness of algorithms when subject to human oversight. AI technologies should indeed be considered as supplements to radiologist practice, increasing physicians’ performance and reducing the scope for human errors (13); clinical decision support applications represent tools which help in diagnosis instead of substitutes of clinicians (14).

In addition to the clinical setting, the use of AI could assist surgeons during intervention (15) and lead to more precise and minimally invasive surgical techniques (16).

The reduction of health professionals’ workload represents another potential advantage of AI (12, 17) and, in particular, in radiology field, Vedantham et al. (12) highlight the potential of AI to improve the timeliness of care through its application in triage phase and in prioritizing the reading list.

The topic of time emerges also from the surveys of Pecqueux et al. (16), in terms of reduction of time spent by specialists on monotonous tasks, and of Emiroglu et al. (9), in terms of possibility to get to a rapid diagnosis and to examine a larger number of patients. Moreover, AI may filter out non-complex cases letting a greater focus of specialists on more challenging cases (18).

AI advantages include also the better epidemiological risk assessment of diseases (19) and the early identification of at-risk patients (20). Beside patients care, AI use is supposed to lower costs (21).

### 3.3 Critical issues

Algorithms’ biases and privacy represent important critical issues of AI use in medicine.

With reference to algorithms using supervised training, Vedantham et al. (12) observe that results may be unreliable when the training phase of the algorithm did not include the encountered data (so-called out of distribution data); related to this aspect, López et al. (17) note that inequalities in provision of healthcare services and low health insurance coverage in low-middle income countries result in a lack of data of certain patients group. The development of predictive AI models which were trained with data that do not reflect the context of use of the algorithm represent the so-called “contextual bias” (17). As consequence, despite the potential to improve the access to care (21, 22), AI algorithms may amplify or create health disparities among marginalized groups (23), augment racial and demographic disparities (13), and exacerbate inequities in health outcomes (18).

Other critical issues deal with the impact on traditional doctor–patient relationship, which dynamic based on a fiduciary model will be challenged (8): patients’ care do not only include the diagnosis but also a “holistic approach with human touch” (22), and the use of AI may result in a low ability to empathize with patients (16). Moreover, the use of AI could result in a less rely on history and medical examination, with a possible decrease in quality of medical care: this also raises questions about legal liability in case of diagnostic errors due to the lack of physical examination (24). Bhardwaj (1) excludes that AI and machine learning will ever substitute “an astute and empathetic bedside clinician.” The topic of substitution relates with another critical issue, consisting in the replacement of doctors’ job and job losses (9–11, 21, 22). The use of AI could moreover affect young doctors’ motivation to try a career as radiologist, with a consequent risk of specialists’ lack, and cause a decrease of training opportunities (25). Further, less experienced clinicians may completely trust the diagnosis of AI algorithms (8).

Another main critical issue is the “black box” phenomenon, that is the “inability to fully understand an AI’s decision-making process and the inability to predict the AI’s decisions or outputs” (26): opacity in decision-making is described (27), and the provision of sufficient evidence about the design, the testing and evaluation can be difficult also for software developers (28). The use of black box systems also raises questions about patients with referral to the principle of autonomy and informed consent (26).

The need to obtain the informed consent before incorporating AI use into patients’ care is highlighted by Mezrich (11), who also highlights the importance to disclose the findings of computer aided detection applications in reports, explaining the reasons of an eventual disagreement with them. According to Jobson et al. (28), clinicians should inform patients of their intention to use AI, and patients should be sufficiently informed to understand risk, benefits and limitations of AI software and give consent to their use. The providing of inaccurate information to patients and clinicians about risks of AI algorithms may indeed represent a breach of the duty of care (28), and adequacy of information provided to users is crucial in judgments (15). However, referring to information, Harvey and Gowda (14) wonders what exactly should be disclosed to patient.

Other critical issues about the use of AI in medicine consist in privacy (1, 8, 11, 13, 17–20, 22, 24, 28, 29) and cybersecurity (18, 29): algorithms’ efficacy is based on large data and some of them may impact on patients’ privacy (22).

### 3.4 Liability issue

Liability in case of AI errors is a main issue and, despite the reviewed articles discuss multiple liability theories in relation to AI use, a unanimous and definitive answer to this issue currently do not exist.

According to Mezrich (11), since AI represents a new technology, the tort law applicable to it is not yet well developed: in case of suit for malpractice, a breach of duty of care and a deviation from the standard of care is required, and, in the context of AI, negligence might derive from a failure in programming, in supervision, from actions of physicians or algorithm itself (11). In case of failure to use or of reliance on AI medical devices, Lang et al. (27) observe that the fault could still be present if best medical practice at that time would have suggested a different approach. Nevertheless, apportioning liability may be difficult especially when using algorithms developed through neural networks, which represent a black box for both the manufacturers and the physicians (24).

Abràmoff et al. (21) note that creators of autonomous AI products should be liable for harms produced by the device if it was used properly and on label, maintaining the responsibility for proper use of the device with the providers; however, in case of assistive AI, in which the clinicians are capable of an independent evaluation of AI outputs, these latter would remain liable. Similarly, Channa et al. (20) state that if autonomous AI is used without specialists through test administration by the primary care physician, who does not have specialized knowledge, this should not be liable for incorrect results and AI creators or the selling company should assume medical liability.

Manufacturing companies should clearly provide the indications and possible adverse effects in using an AI algorithm (8), and, according to Bazoukis et al. (18), if there are potential adverse events/system failures attributable to the use of algorithms, the developers of augmented intelligence should report it and investigate outcomes, narrowing down clinicians’ responsibility to the “labeled” use of algorithms.

Furthermore, defining the degree of control is of primary importance when discussing liability issue: according to Sung and Poon (15), when machines, based on self-learned algorithm, achieve diagnosis without need of clinician’s approval, liability should at least partially be considered of the AI developer; if fully automated surgery is used, events produced by defects in configuration or design would probably be a responsibility of developers in a product liability claim. The same authors, however, affirm that the developers should not be considered liable just because their AI devices are not able to prevent harm in all instances (15). Even Mezrich (10) observes that the legal handling of AI will be based on the degree of autonomy of the software: when AI is used only as a decision support, the radiologist who makes the final determination bears the liability risk. Differently, if AI algorithm acts as a subordinate of the radiologist, the doctrine of vicarious liability could be applied (10). The model of vicarious liability, which provides that the negligence of an assistant is attributed to the supervisor (“respondeat superior”), is discussed by multiple authors (10, 11, 13, 14, 25). According to Mezrich (10, 11), as an AI algorithm which acts autonomously could be considered analogous to an employee of a facility, its negligence could be attributed to its supervising radiologist or to the institution; according to Harvey and Gowda (13) in clinical settings, hospitals, and physician groups would be responsible for the malpractice of their employees’ use of AI programs. Neri et al. (25) hypothesize an AI subjected to a vicarious civil liability, “written in the software and for which the producers must guarantee the users, so that they can use AI reasonably and with a human-controlled automation.”

Another discussed model consists in the product liability, which requires the existence of a defect in the product and provides that the manufacturer of the defective product is presumed to be responsible of this latter (27): according to Mezrich (10, 11) this model could be obstacled since the original developed algorithm may not be identical to the one that causes the harm, as it improves itself over the time. While explaining this model, Mezrich (10, 11) refers also to the learned intermediary exception, according to which a radiologist would be responsible for liability if he/she has had the chance to review the report and detect errors before its releasing and before the patient was injured. The same author observes that additional aspects of the product liability may derive from the development of AI software by an external vendor and its training “in-house” by a radiology department: in this case, enterprise liability would apply, with a consequent possible liability also of the customizing facility. With reference to the learned intermediary doctrine, Nitiéma et al. (24) note that patients are prevented from directly suing medical device manufacturer and the prescriber represent the end user of the device.

Nitiéma et al. (24) highlight that one proposed legal solution to liability issue consist in the conferral of personhood to AI devices, that would result in direct lawsuits against them in case of malpractice. The conferral of personhood to AI algorithms is also considered by Mezrich (10, 11). Moreover, Mezrich (10, 11) refers to the model of the vaccine injury compensation program, as a possible approach. With regard to AI applications in surgery, a cooperative effort of surgeons and assistive AI systems is hypothesized by Pecqueux et al. (16).

Chan (26), after deeply analyzing different theories of liability, proposes that physician, manufacturers and hospitals are considered a common enterprise for the purposes of liability: such approach “shift away from individualistic notions of responsibility, embodied by negligence and products liability, toward a more distributed conception.” The same author affirms that “a common enterprise strict liability approach would create strong incentives for the relevant actors to take care.”

## 4 Discussion

Artificial intelligence refers to software or programs able to perform operations similar to the human activity of learning and decision-making through the use of technologies based on processes of machine learning, deep learning, and neural networks (30). The use of AI in medicine today represents an emerging reality with increasingly clear and secure lines of development. If the advantages that the contribution of automated medicine triggers in the patient’s healthcare and protection system are certain, the implications on a legal and liability level that derive from AI tools are less certain (7). At the root of the problem there are first of all the characteristics of the tool, which does not allow the operator to access the processes through which it achieves the desired result (31). When analyzing the nature of the AI background, it is necessary to keep in mind a gradual transition that it is already making in many areas of its use, that from automation to autonomy: while the automaton performs controlled and controlled by humans (so-called machine learning), the autonomous AI system, or with an increasingly greater degree of autonomy, is able to process the data it collects in a completely independent way, and to act in the context that surrounds it by system that data. The result is a behavior that, net of a desired result achieved, is not knowable by man, not even by the one who produced it (so-called deep learning). The processes that the machine puts in place, in fact, are difficult to identify ex post, as well as ex ante, in the sense that the operator can count on the relative certainty of the result that the machine will complete, but will not be able to identify the “how” of that procedure. The result is a system characterized by complexity, incompleteness, opacity, unpredictability, openness and vulnerability. These characteristics undermine the current models of responsibility, in particular, those that are based on the criterion of imputation of fault and on its identification for the purposes of liability and compensation.

Literature review highlights that issue of liability in case of AI-related error has received growing attention in recent years, nevertheless this issue is mainly discussed in position papers and non-systematic reviews, and more in-depth analysis are scarce. In line with the above, it has emerged that difficulties in apportioning liability are present especially when algorithms developed through neural networks are used, as they cannot be fully understandable both for manufacturer and clinicians, constituting a black box (24). Since AI algorithms’ lack of explainability, it could be difficult for doctors to assess whether the diagnosis or recommendations of an AI system are sound in relation to their own knowledge (26). Moreover, unexperienced physicians may blindly trust diagnosis of AI algorithms so that, according to Chung et al. (8), “a complication or medical malpractice may be further perplexed, since both healthcare professionals and AI developers are involved.”

Another issue related to this aspect consists in the possible presence of biases during the development phase of AI algorithms, which may lead to the incorrect identification of an ill person as healthy (underdiagnosis) and result in a delayed access to care (32). Automatic machine learning requires indeed “big data” to instruct algorithms, yet sometimes these latter are affected by unsatisfactory training samples with repercussions on generalization (33). Alongside clinical consequences, similar situations may raise claims difficult to resolve. The impossibility to identify the exact moment in which the error has occurred, would indeed prevent the finding of the link between the error and the damage, and preclude the compensation. One could, as an evaluation criterion, refer to the degree of autonomy of AI to identify the professional’s contribution: when it is minimal and therefore AI is completely autonomous even in its updates or when human intervention is essential. According to Sung et al. (15), when clinicians or developers are capable to prevent an accident, legal liability is likely to be defined, whereas in case of diagnosis and treatment achieved basing on self-learned algorithms which do not require clinicians’ approval, responsibility should be at least partially attributed to the AI developer. Usually, however, AI as a calculation program used in health systems and hospitals needs a human contribution through which the definitive result is reached, and therefore it is important to analyze the responsibility separating the various profiles.

The use of AI algorithms in medicine indeed implies multiple actors, consisting in developers, hospitals, physicians. Negligence could be expression of a failure of programming, supervision, clinicians’ actions or of the algorithm (11). Therefore, it would be important to differentiate when the error can be traced back to the doctor for the improper use of these systems or when it is only and exclusively attributable to the hospital that manages and sets the instrument that does not require additional human aids. It would also be important to trace the consent of patients to the use of new technologies in clinical practice (33). The information given to patients about risks, benefits and limits of AI is a step of paramount importance, and it should be aimed at ensuring full awareness of choices and eventual alternative pathways in case of resistance to new technologies (34). In this context, however, the difficulty of explaining how AI systems transform inputs into outputs represent a challenge for the informed consent, and sometimes patients’ level of awareness could be not sufficient to make this latter possible. Clinicians could be considered as mediators between patients and AI systems, being the recipients of an explanation from the AI system and acting toward the patient as a translator into a meaningful and easily understood format (35). As pointed out in other medical contexts, doctors have a professional duty to explain and discuss risks, benefits and possible alternatives to a procedure: the information process should take into account the fact that patients may not be able to assess the risks in abstract terms and they may therefore need to rely on a comparison to achieve an informed decision (36). Moreover, the informed consent is supposed to be achieved through an appropriate legal framework for data protection: the machine learning processes require large amounts of data whose right of use is often differently regulated among countries, especially with regard to the degree of de-identification of the patient’s identity (37). The issue of consent is also crucial in view of the progression toward personalized medicine, which is an area in which some fundamental problems of the use of AI in medicine re-emerge, namely privacy, stratification and discrimination of sub-populations on the basis of ethnicity, equality of access and fair allocation of resources (38).

Already in 2018, the European Commission decided to launch a coordinated plan on AI with European Union Member States (in addition to Switzerland and Norway), and in 2019 it published ethics guidelines for trustworthy AI, which emphasized the importance of AI in healthcare with reference to the ageing of the European population. Similarly, the World Health Organization issued in 2021 a guidance on Artificial Intelligence in Health including principles for its design and use (37). In September 2022, a proposal for a new directive on liability of defective products and a proposal for a directive on adapting non-contractual civil liability rules to AI were published by the European Commission (39, 40). This latter purposes to “improve the functioning of the internal market by laying down uniform requirements for non-contractual civil liability for damage caused with the involvement AI systems,” and it would create a “rebuttable presumption of causality,” in order to simplify the burden of proof for harmed people. Despite the provision of some uniform liability rules, however, according to Duffourc et al. (41) by applying these directives, potential liability gaps would persist when using some black-box medical AI systems.

The reviewed literature analyses the current theories of liability, trying to apply them to AI context, including medical malpractice, vicarious liability, product liability, learned intermediary exception. Briefly, according to malpractice law, the physician could be considered liable in negligence “for harmful medical errors that fall below the standard of care” (26). According to the vicarious liability model instead the faults of subordinates would be in charge of principals, for example the hospitals or physician groups in clinical settings (13). Product liability relates to the existence of a defect in the product, and learned intermediary doctrine identifies the physician as intermediary (10, 11, 26). In the context of AI, giving AI personhood has been discussed as a possible solution, so that harmed patients could directly sue AI devices (10, 11, 24). Chan (26) instead suggests a common enterprise model, which encloses manufacturers, physicians and hospitals, which would let a shift from the individualistic concept of responsibility toward a more distributed one.

In authors’ opinion, it would be interesting to think of the relationship between the hospital and AI as that of an institution that, by making this “diagnostic assistant” available, behaves in the relationship with this instrument as a supervisor, as a custodian, which guarantees in the hospital environment its correct use. It is also interesting to note that the uncertainties in the area of professional liability are not only related to AI algorithms, but have also previously arisen referring to the use of telemedicine, for which some of the hypothesized profiles were similar: production defects of the equipment, omitted/defective/ineffective maintenance, errors in the use of the equipment (42). In that context, not only have multiple normative acts, projects and strategic documents been implemented at European level, but also at national level policies aimed at increasing the diffusion and efficiency of telemedicine services (including between 2014 and 2021, the National Guidelines for Telemedicine, the Digital Growth Strategy, the National Directives for the Delivery of Telemedicine Services, and the National Recovery and Resilience Plan) (43). That said, it is clear that the increasing use of AI in medicine will lead to legal challenges regarding medical negligence and that for this reason on the one hand the legal system should provide clear indications on what entity holds the liability, and on the other hand insurances should make explicit the terms of coverage in the event that healthcare decisions are partially achieved an AI system (44).

In any case, the result is a real revolution in the relationship between the patient and the physician, who was born as a pater and undisputed mentor of the patient in the paternalistic model, further evolved into an ally in the management of the patient’s therapy in the model of reciprocity, and is undergoing a new evolution due to the intervention of a third element in the relationship: the AI. This evolution, which is going to challenge the fiduciary doctor–patients relationship as it is known, opens up new medico-legal scenarios in the area of liability. The consequence of the increasing use of automatic technology in healthcare could therefore be a new conception of the doctor as human in command, which sometimes would only keep the role of controlling the final result of the machine (output) and validating it. In such a context, it is conceivable that in case of default on the part of AI intelligence, of which the hospital act as custodian and owner, it will be the latter that with the professional will have to answer, and the hospital will have to provide for a specific insurance that covers that particular risk with a specific “diagnostic assistant” or “algorithm liability” liability insurance contract.

One of the most well-known critical points of legal regulations consists in the difficulty of adapting to new realities. A regulation is considered evolved the more it manages to capture in its own meshes events that are dissimilar to the typical norm that may occur in the future and which can be subsumed by it: this can only happen if it includes broad definitions and at the same time clear, precise, and broad prescriptions. In the current context, in order to answer to the issue of liability for the use AI algorithms, it is essential in the legislative production a scientific as well as legal preparation, since it engages the law in forms of protection not yet known, prompted by events that only science can foresee.

## 5 Conclusion

The increasingly widespread use of intelligent systems, capable of learning and making decisions, opens up new exciting frontiers, but at the same time, it radically changes the relationship between humans and technology. Over the last 3 years, the interest about advantages and critical issues related to the application of AI in medicine has significantly grown, with an increasing attention to the potential liability consequences in case of error. Alongside its potentials, the wide use of AI still raises concerns regarding possible development bias, disparities, informed consent, privacy, doctor–patient relationship, and liability. Since the use of AI in healthcare involves multiple actors, consisting in manufacturer, hospitals, physicians, different solutions to the liability issue have been analyzed, which however do not identify a definitive answer. The application of AI is rapidly extending to all healthcare setting, and an unequivocal answer on the allocation of responsibility in case of errors is becoming increasingly necessary. The danger of over-reliance and excessive dependency on such systems, which could lead to significant effects of de-skilling and desensitization of doctors to the clinical context, should also be highlighted and studied. It is also necessary for AI systems to explain to operators how they arrived at their conclusion and decision, providing the evidence that underlies their reasoning. This way, they can potentially choose to refuse to follow the suggestion if they believe any errors might have been made. In order to outline a reference regulatory framework to which address issues arising from the use of AI algorithms, broad definitions but at the same time clear and precise prescriptions should be provided based on scientific evidences.

## Author contributions

CC: Data curation, Formal analysis, Investigation, Methodology, Writing – original draft. AD: Data curation, Software, Writing – review and editing. BM: Data curation, Software, Writing – review and editing. LC: Conceptualization, Writing – review and editing. PT: Conceptualization, Supervision, Writing – original draft.
